# Attitudes and satisfaction in the care of chronically ill children: toward an integrated model of family and professional support

**DOI:** 10.3389/fpubh.2026.1789430

**Published:** 2026-05-07

**Authors:** Beáta Erika Nagy, Péter Boris, Karolina Eszter Kovács

**Affiliations:** 1 Pediatric Rehabilitation, Pediatric Psychology and Psychosomatic Unit, Faculty of Medicine, Institute of Pediatrics, University of Debrecen, Debrecen, Hungary; 2 Laki Kálmán Doctoral School, University of Debrecen, Debrecen, Hungary; 3 Faculty of Arts, Institute of Psychology, University of Debrecen, Debrecen, Hungary

**Keywords:** attitudes toward chronic illness, child and adolescent mental health, childhood chronic illness, family–school–healthcare collaboration, parental satisfaction, psychosocial wellbeing

## Abstract

**Background:**

Childhood chronic illness affects not only medical outcomes but also psychosocial wellbeing, requiring coordinated support from families, schools, and healthcare systems. While patient satisfaction and attitudes toward chronic illness have been studied separately, less is known about how these perspectives interact across key reference groups.

**Methods:**

A cross-sectional quantitative study was conducted among children aged 10–18 years with chronic illnesses and their reference persons in a university paediatric clinic. The sample included 107 children and their parents, along with teachers and healthcare professionals. Patient and parental satisfaction with care were assessed using a structured satisfaction questionnaire, while attitudes toward chronic illness among parents, teachers, and healthcare workers were measured across educational, social/community, and future-oriented dimensions. Factor analysis, cluster analysis, correlation analyses, and regression models were applied to explore satisfaction patterns, attitudinal differences, and interrelationships.

**Results:**

Three distinct parental satisfaction profiles were identified: parents satisfied with all aspects of care, parents dissatisfied with disease-specific information, and parents dissatisfied with examinations and information. Higher parental satisfaction was associated with better child psychosocial outcomes, including lower illness burden and higher health-related quality of life. Attitude profiles differed across reference groups: parents showed the strongest educational attitudes but more negative perceptions of community inclusion and future prospects, whereas teachers and healthcare professionals demonstrated more positive community-focused and future-oriented attitudes. Intergroup analyses revealed meaningful associations, particularly in future-oriented attitudes, which were positively related to parental satisfaction.

**Conclusion:**

The findings suggest potentially complementary roles of parents, teachers, and healthcare professionals in supporting children with chronic illness. Strengthening cross-sectoral collaboration and aligning attitudes across family, educational, and healthcare contexts may enhance patient satisfaction and promote psychosocial wellbeing.

## 1 Introduction

The experience of chronic illness in childhood poses profound challenges not only for the affected children but also for their families, educators, and healthcare professionals (1–4). Children with chronic diseases often face disruptions in everyday life, emotional strain, and academic difficulties, while their parents experience increased psychological burden and must coordinate complex medical and educational support systems (5). Understanding the satisfaction and attitudes of these key reference persons is therefore critical for enhancing both the quality of care and the child’s overall well-being (6, 7).

Internationally, the movement toward patient- and family-centered care (PFCC) emphasizes empathy, communication, and shared decision-making as foundations of effective healthcare (8). Beyond medical outcomes, care must address the individual needs, emotions, and values of patients and families. Studies have shown that parental satisfaction in paediatric settings is closely linked to physicians’ availability, empathy, information sharing, and consistency of care (9, 10). Likewise, children’s own satisfaction with the hospital environment and their interactions with caregivers can influence their adjustment, resilience, and recovery.

Parallel to these developments, research on attitudes toward chronic illness highlights that parents, teachers, and healthcare professionals all play a formative role in shaping a chronically ill child’s coping and self-perception (11). Parents’ attitudes influence daily adaptation and treatment adherence; teachers’ perspectives affect inclusion and educational participation; and healthcare workers’ empathy and communication shape therapeutic collaboration. However, existing studies have largely examined parental satisfaction and attitudes toward chronic illness in isolation, with very limited attention to how these perspectives interact across key reference groups within the child’s microsystem (family, school, and healthcare). As a result, there is a lack of integrated evidence on how attitudes across these domains relate to parental satisfaction and child psychosocial outcomes within a shared relational context (12).

The treatment environment plays a pivotal role in the healing process, particularly for children. In paediatric care, the active involvement of families and parents in the therapeutic process is especially significant. Individuals living with chronic illnesses and their families generally identify three key elements of effective patient- and family-centered care (11):

1. the establishment of emotional and social connection between patients and healthcare professionals,
2. a sense of empowerment that enables patients and family members to take part in the care process, and
3. the perception that the care provided is genuinely effective.

Parents’ attitudes toward their child’s chronic condition reflect their emotional reactions, coping mechanisms, and beliefs about how the illness influences the child’s daily functioning and the family’s overall dynamics. Likewise, healthcare professionals’ approaches to chronic illness are shaped by their underlying beliefs, emotional engagement, and behavioral intentions toward affected patients, all of which can influence the quality of care and support offered (13).

In addition to family and medical involvement, the role of teachers is also fundamental, as children with chronic health conditions frequently face educational challenges that affect both academic and social development. Teachers’ perceptions and emotional attitudes toward students with chronic illnesses—together with their willingness to provide suitable accommodations—have a decisive impact on the inclusivity and effectiveness of the educational environment (12).

To address this gap, the present study adopts an integrated theoretical framework in which parental satisfaction, attitudes toward chronic illness, and child psychosocial outcomes are conceptually linked. Drawing on patient- and family-centered care (PFCC), parental satisfaction is interpreted as a key indicator of perceived quality of care, reflecting communication, involvement, and emotional support (8). From an ecological perspective, attitudes toward chronic illness held by parents, teachers, and healthcare professionals represent proximal environmental influences within the child’s microsystem (14). These attitudes shape expectations, social inclusion, and coping opportunities. Within this framework, parental satisfaction is assumed to be influenced not only by healthcare experiences but also by the broader attitudinal climate surrounding the child (15). In turn, both satisfaction and attitudes are expected to contribute to children’s psychosocial wellbeing, including quality of life, emotional functioning, and perceived illness burden (16, 17). In the present study, attitudes toward chronic illness are conceptualised primarily as contextual and relational factors within the child’s microsystem, rather than strictly causal antecedents. At the same time, they are assumed to function as proximal predictors of parental satisfaction and child psychosocial outcomes, shaping expectations, communication patterns, and perceived support. Given the cross-sectional design, these relationships are interpreted as associative rather than causal.

Beyond addressing this gap, the present study offers a novel contribution by jointly modeling parental satisfaction and attitudes toward chronic illness across multiple reference groups. While previous research has typically examined parental satisfaction or professional attitudes in isolation, the current approach captures their interrelated roles within a shared psychosocial system. This integrated perspective allows for the identification of cross-contextual patterns, highlighting how attitudes in the family, school, and healthcare domains align or diverge, and how these patterns relate to parental satisfaction and child wellbeing. In doing so, the study moves beyond single-domain analyses and provides a more comprehensive understanding of the relational dynamics shaping the experience of childhood chronic illness. This approach contributes to the literature by reframing parental satisfaction not only as an outcome of care, but as a construct embedded within a broader attitudinal ecosystem.

Therefore, the present study integrates these two strands of inquiry by examining (1) patterns of patient and parental satisfaction with paediatric care, and (2) the attitudes of parents, teachers, and healthcare workers toward chronic illness. Through a cross-sectional, quantitative design, the research aims to identify the psychosocial and attitudinal factors that support higher patient satisfaction and to explore a preliminary attitude model describing how reference persons may be interrelated in relation to children’s wellbeing.

In the research, we had the following research questions:

*RQ1*: What are the key dimensions of patient and parental satisfaction in paediatric chronic care, and how do these dimensions interrelate?

*RQ2*: Are there distinct groups of parents based on their satisfaction levels with paediatric healthcare (e.g., satisfied, dissatisfied with information, dissatisfied with examinations)?

*RQ3*: How are parental satisfaction and children’s satisfaction with hospital care related?

*RQ4*: How do the attitudes of parents, teachers, and healthcare workers toward chronic illness differ across educational, social/community, and future-oriented dimensions?

*RQ*5: How are these attitude dimensions interrelated across the three reference groups, and how do they predict parental satisfaction and perceived child wellbeing?

## 2 Methods

### 2.1 Study design and participants

A cross-sectional quantitative study was conducted to explore patient satisfaction and attitudes toward chronic illness among children aged 10–18 with chronic diseases and their reference persons (parents, teachers, and healthcare professionals) (Table 1). Participants were recruited from the gastroenterology, pulmonology, onco-haematology, and rehabilitation departments of the University of Debrecen Paediatric Clinic. In the overall study, the study group consisted of 300 children and caregivers (294 children and parents, 588 in total), of whom 107 sick children and parents had their questionnaires analysed (from the current analysis, healthy control group has been excluded). Inclusion in the final analytical sample required the availability of complete and matching data across key variables, including parental satisfaction, child psychosocial measures, and at least one set of attitude assessments. Each child received a unique identification number to ensure anonymity. Participation was voluntary, and all respondents completed the questionnaires in person under the supervision of a clinical psychologist. It is also worth mentioning that the COVID-19 pandemic caused by the SARS-CoV-2 virus, a pandemic of COVID-19, also made it challenging to take tests on several occasions when people wanted to spend the minimum time in and around the clinics. Once they had undergone the necessary medical examination, they tried to leave immediately. Children with intellectual disabilities or illiteracy were excluded.

**Table 1:** Sociodemographic characteristics of the study sample.

Characteristics	*N*	%
Gender		
Boy	58	54.2
Girl	49	45.8
Residence		
County seat	48	44.9
Big city	12	11.2
Small town	28	26.2
Village	19	17.7
Mother’s education		
Primary	21	19.6
Secondary	59	55.4
Tertiary	27	25
Father’s education		
Primary	23	21.8
Secondary	65	60
Tertiary	19	18.2
Financial situation		
Low	85	84.5
Average	11	12.1
High	11	12.1
Patient group		
Gastroenterology	30	28.0
Onco-haematology	26	24.3
Pulmonology	18	16.9
Rehabilitation	33	30.8

Based on the compiled demographic questionnaire, a total of 107 individuals participated in the study. The gender distribution consisted of 58 boys (54.2%) and 49 girls (45.8%). The average age of the participants was 14.3 years, with a standard deviation of 2.0. In terms of residency, 48 individuals (44.9%) lived in the county seat, 12 (11.2%) in the big city, 28 (26.2%) in small towns, and 19 (17.7%) in villages. This distribution was intentionally designed to ensure a diverse representation of opinions, not solely from the county town population. When considering birth order, one participant had no siblings, 63 were first born, 26 were second born, and the remaining 17 were the third or later child in their families. Table 1 provides a comprehensive overview of the sociodemographic characteristics of the sample.

The majority of the participants, specifically 83.8%, reside in their parents’ household. A smaller percentage, 9.1%, live with their mother, while an even smaller portion, 3%, live with their father. Additionally, 1% of the respondents live with a grandparent, and 3% reside with a foster parent. Concerning the living arrangements, a significant number, 80.8%, have an own room, while the remaining 19.2% do not currently possess one. Among those with a room, the majority, 78.8%, sleep in their own space. However, 11.1% share a room with their parents, and 10.1% share a room with their siblings. In terms of financial standing, the subjective evaluation provided by the participants indicates that 84% consider themselves to be living on an average income. Conversely, 13% believe they are living on a low income, while the remaining 3% claim to have a high income.

The breakdown of patients (specifically children) was as follows: 34 (16.4%) with gastroenterological, 21 (10.1%) with onco-haematological, 19 (9.3%) with pulmonological, and 33 (15.9%) with rehabilitation-related disorder. A majority of 82 individuals (76.64%) reported that none of their immediate family members had a chronic illness. In contrast, 25 individuals (23.36%) disclosed that there were family members, both children and adults, who suffered from chronic diseases.

### 2.2 Instruments

To ensure conceptual clarity, the psychological measures were selected to capture key domains of child psychosocial wellbeing, with particular emphasis on health-related quality of life, illness burden, and emotional functioning as primary outcomes. Additional measures (e.g., resilience, life satisfaction, and behavioral indicators) were included to provide a broader contextual understanding of the child’s psychological profile.

With children and adolescents, we took a pre-assembled set of tests to assess the components of the personality profile of children with chronic illnesses that might be related to our study objective in the reviewed literature. In our self-designed Sociodemographic Questionnaire, we formulated questions regarding gender, age, type of residence, parents’ highest educational level, family structure, subjectively assessed financial status, number of siblings, exact description of chronic disease(s), and achievement of independent (alone) sleep. In addition, the following psychological measures were used:

- The Hungarian version of the Connor-Davidson Resilience Questionnaire (18) measures successful coping with stress through a factor, scoring between 10 and 40 points.
- The Satisfaction With Life Scale (SWLS) (19) and the Cantril Ladder (20) measure subjective well-being.
- The EuroQol 5 Dimensions Youth 5 Level (EQ5DY) scale (21) measures health-related quality of life in children 8 years and older.
- The Non-Productive Thoughts Questionnaire (NPG-K) is a single-factor scale (22) for measuring ruminations and rumination in childhood, with scores ranging from 10 to 30.
- The Problematic Internet Use Questionnaire (PIU-Q) for adolescents, an abridged version (23) measures children’s and young people’s attitudes toward the Internet on three subscales: preoccupation with Internet thinking (obsession), neglect of daily activities (neglect) and difficulty controlling Internet use (control).
- In the Drawing version of Pictorial Representation of Illness Self-Measure, PRISM-D (24), four subscales of the drawing test developed by the Hungarian working group of the PRISM test (25) were considered: the distance between the yellow circle symbolizing self and the circle symbolizing illness (SIS), the average area of the circle representing illness 25.12 cm2 (IPM), the number of circles representing youth resources (NC), and the total area of the circles representing resources (AC) were compared.
- The Beck Depression Inventory—Shortened Scale [BDI—R (26, 27)], the most reliable measure of depressive symptom severity
- The Illness Intrusiveness Ratings Scale [IIRS (28)], a measure of “illness burden” as a means of assessing the impact of chronic illness and its treatment on different aspects of life
- The Spielberger State—Trait Anxiety Questionnaire—Child Version (29) were developed based on the adult questionnaire of the same name to assess anxiety levels in school-age children.
- The Strength and Difficulty Questionnaire [SDQ (30, 31)] measures children’s behavioral characteristics and abilities and provides a consistent picture compared to the parent version.
- A Patient Satisfaction Questionnaire (own edition) assessed satisfaction with general and disease-specific information, facilities and cleanliness, and examination condition. The questionnaire consists of 30 items rated on a 5-point Likert scale (ranging from 1 = strongly disagree to 5 = strongly agree), with higher scores indicating greater satisfaction. The items cover key aspects of care, including general information, disease-specific information, communication with healthcare professionals, examination conditions, and environmental factors (e.g., facilities and cleanliness).

- For parental satisfaction, satisfaction with general information, satisfaction with disease-specific information, satisfaction with facilities and cleanliness, and satisfaction with the conditions of the study (KMO = 0.614; *χ*^2^ = 270.290; df = 36; *p* < 0.001).
- Satisfaction with information, satisfaction with facilities and cleanliness, and satisfaction with the conditions of the examination (KMO = 0.633, *χ*^2^ = 274.562; df = 78; *p* < 0.001)
- For parents, teachers, and healthcare professionals, the Chronic Illness Attitudes Questionnaire (CIAQ) (32) measured attitudes across three dimensions: social relationship and community attitudes (14 items), educational attitudes (13 items), and vision for the future (7 items). The CIAQ showed acceptable internal consistency (Cronbach’s *α* = 0.65–0.73 across groups), although some subscales demonstrated modest reliability, which should be taken into account when interpreting the results. Given the number of variables assessed, the analyses were guided by theoretically informed hypotheses, and emphasis was placed on consistent patterns rather than isolated significant findings.

### 2.3 Statistical analysis

To enhance clarity and transparency, each analytical step was aligned with the specific research questions. Factor analysis was used to address RQ1 by identifying the underlying dimensions of patient and parental satisfaction. Cluster analysis was applied to RQ2 to explore potential patterns of parental satisfaction. Correlation analyses were conducted to examine associations between parental and child satisfaction (RQ3). Group differences in attitudes across parents, teachers, and healthcare professionals were tested using non-parametric methods (RQ4). Finally, regression analyses were performed to assess the extent to which attitude dimensions predict parental satisfaction and child psychosocial outcomes (RQ5).

Data were entered into Excel and analysed using IBM SPSS 22.0 and Jamovi 2.3.28. For patient satisfaction, factor analyses (maximum likelihood, Varimax rotation) identified satisfaction components. K-means cluster analysis (100 iterations) was applied as an exploratory method to identify potential patterns of parental satisfaction. This approach was chosen due to its suitability for grouping individuals based on continuous variables and its interpretability in applied psychological research. Given the sample size, the analysis was conducted with caution and aimed to provide an initial typology rather than definitive classification. ANOVA, chi-square tests, and Pearson’s correlations examined relationships among sociodemographic, psychological, and satisfaction factors. For attitude analyses, Kruskal–Wallis and Mann–Whitney U-tests were applied due to non-normal data distribution, and Spearman’s rank correlations tested inter-group relationships. Linear regression models were conducted to assess predictors of parental satisfaction.

Child psychosocial outcome variables were analysed separately rather than combined into a composite score, in order to preserve their conceptual distinctiveness. These variables were primarily used in group comparisons and correlational analyses examining associations with parental satisfaction.

### 2.4 Ethics

The study was approved by the Institutional Research Ethics Committee of the University of Debrecen Clinical Centre (DE RKEB/IKEB 5858–2021) and followed the Declaration of Helsinki. Informed consent was obtained from parents or legal guardians of minors.

## 3 Results

### 3.1 Patient and parent satisfaction patterns

The analysis of parental satisfaction revealed three clusters: (1) Parents satisfied with all aspects of care (*N* = 83)—consistently positive ratings across all satisfaction components; (2) Parents dissatisfied with disease-specific information (*N* = 5)—negative attitudes toward information provision but neutral or slightly positive on other factors; (3) Parents dissatisfied with examinations and information (*N* = 14)—negative views of examination conditions and general information, but moderate satisfaction with facilities and cleanliness. However, it should be noted that one of the clusters was relatively small (*N* = 5), which warrants cautious interpretation.

**Figure 1:**
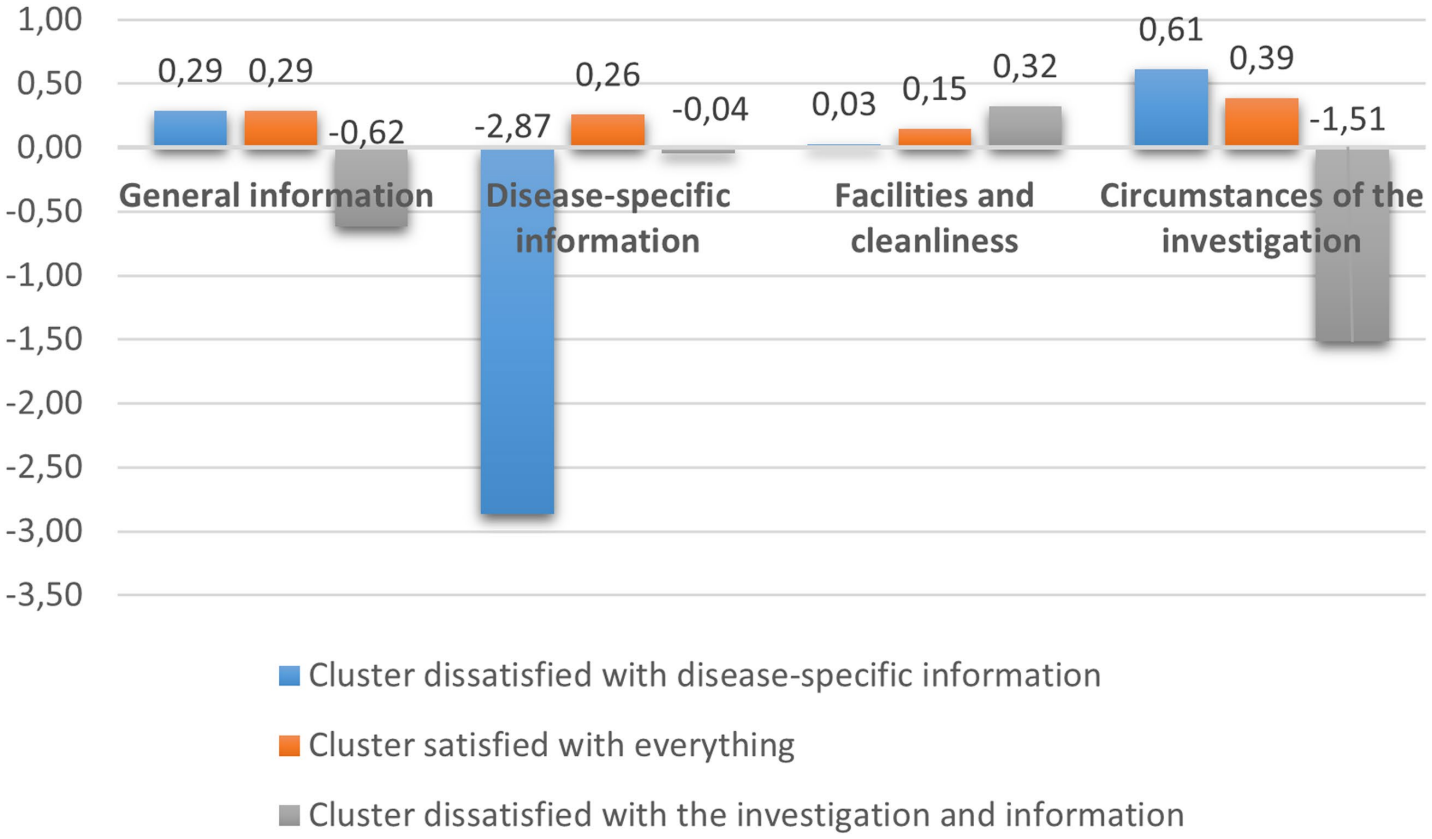
Parent groups based on patient satisfaction.

The central values of the clusters is presented in Figure 1.

No significant differences were observed between clusters regarding sociodemographic characteristics. Children of satisfied parents tended to show lower illness burden, fewer depressive symptoms, and higher health-related quality of life and peer relationship scores compared with children of dissatisfied parents; however, these differences should be interpreted with caution given the uneven cluster sizes.

Comparison across parental satisfaction clusters revealed no statistically significant differences in most child psychosocial outcomes, with the exception of perceived illness burden (IIRS_total), which showed a significant difference (*p* = 0.015). Although more favorable trends were observed among children of fully satisfied parents—such as lower levels of depressive symptoms and better quality of life—these differences did not reach statistical significance and should therefore be interpreted with caution (Table 2).

**Table 2:** Characteristics of the clusters.

Cluster number of case	Dissatisfied with disease-specific information		Satisfied with everything		Satisfied with the investigation and information		*p*
	*M*	SD	*M*	SD	*M*	SD	
Satisfaction with life	31.20	3.03	28.37	5.15	30.29	4.92	0.236
Cantril ladder	6.40	1.95	6.56	2.16	7.64	2.24	0.218
Resilience	31.60	3.58	29.10	6.57	29.00	6.63	0.699
Non-productive thoughts	18.80	6.06	19.41	4.60	21.43	4.89	0.309
EQ5DY_1	5.40	0.55	6.07	1.78	7.14	2.07	0.075
EQ5DY_2	78.00	16.43	84.48	16.33	77.14	16.43	0.235
Problematic internet use—obsession	3.20	1.64	3.46	1.84	3.14	1.17	0.798
Problematic internet use—abandonment	6.40	2.07	5.04	1.97	4.43	1.87	0.159
Problematic internet use—control	4.20	1.30	3.82	1.86	4.71	2.16	0.253
PRISM SIS	11.30	4.09	11.77	17.14	9.11	6.59	0.843
PRISM IPM	9.10	4.54	21.68	81.41	14.02	16.69	0.891
PRISM number of circles	4.40	1.34	2.86	1.82	3.14	2.03	0.184
PRISM area of circles	111.37	112.81	650.69	5019.88	150.69	343.95	0.908
BDI-R	10.40	1.52	12.43	4.16	14.79	5.74	0.089
IIRS_total	27.00	13.77	18.61	10.48	26.64	11.91	0.015
IIRS_relationship	9.20	6.10	8.73	5.57	10.21	5.92	0.660
IIRS_Int	3.80	3.49	2.77	2.26	2.57	1.40	0.562
IIRS_tool	14.20	5.17	11.04	7.86	13.79	6.69	0.340
STAI-state	45.20	18.20	45.08	11.74	46.64	12.98	0.907
STAI-trait	33.20	6.83	30.41	7.38	33.00	9.24	0.398
SDQ-Hyp	5.20	0.84	4.33	1.90	5.36	1.55	0.107
SDQ-Érz	2.00	2.12	2.55	2.54	3.29	2.81	0.526
SDQ-Vis	1.40	0.89	2.20	1.41	2.57	1.40	0.273
SDQ-Kort	4.00	1.41	5.02	1.32	4.29	1.44	0.057
SDQ-ProSz	8.60	1.52	7.76	1.67	8.57	1.50	0.152

Significant differences were also found in attitudes toward special needs groups, with satisfied parents scoring higher in peer and community attitudes (*p* = 0.028) and future vision (*p* = 0.021). These parents tended to perceive stronger social support, more positive community engagement, and optimism regarding their child’s future development. Correlation analyses demonstrated positive associations between parent and child satisfaction, particularly between parental satisfaction with disease-specific information and children’s satisfaction with examination conditions (*r* = 0.306, *p* = 0.002) (see Table 3).

**Table 3:** Correlations between child and parental patient satisfaction.

Clusters	Statistics	Information for children	Conditions for children - Facilities and cleanliness	Children’s circumstances - the circumstances of the study
General information for parents	r	0.026	0.158	−0.034
	p	0.793	0.112	0.738
	N	102	102	102
Disease-specific information for parents	r	0.062	0.208^*^	0.306^**^
	p	0.539	0.036	0.002
	N	102	102	102
Parental equipment and cleanliness as a condition	r	0.052	0.300^**^	−0.041
	p	0.609	0.002	0.686
	N	101	101	101
Parent inquiry circumstances	r	−0.027	0.072	0.070
	p	0.789	0.474	0.486
	N	101	101	101

*p<0.05; **p<0.01.

### 3.2 Attitude model of reference persons

**Figure 2:**
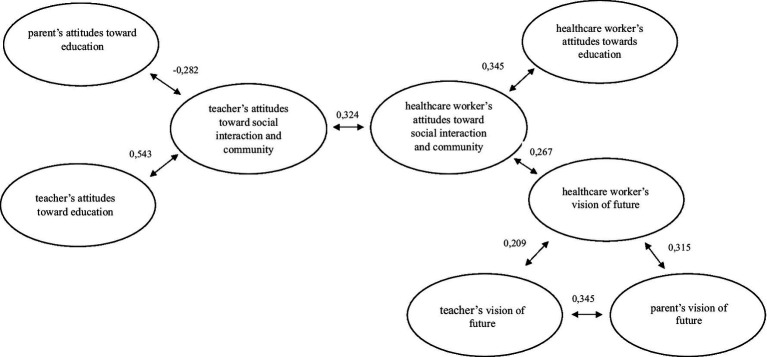
Model of children’s reference persons’ attitudes toward chronic illness.

Comparative analyses of parents, teachers, and healthcare workers revealed distinct attitude profiles. Parents scored highest in educational attitudes, reflecting their central caregiving role but also more negative perceptions regarding community acceptance and the child’s future. Teachers showed the most positive social and community attitudes and the most optimistic vision of the future, indicating strong engagement and belief in long-term development. Healthcare workers demonstrated positive community and visionary attitudes, aligning with patient-centered and preventive care perspectives. The attitude model, interpreted as a pattern of associations among reference persons’ perspectives, showed that teachers’ positive community attitudes were negatively correlated with parental attitudes (*r* = −0.282, *p* = 0.035) but positively associated with healthcare workers’ community attitudes (*r* = 0.324, *p* = 0.002). A consistent positive link across all groups was found in future vision, particularly between healthcare workers and parents (*r* = 0.315, *p* = 0.003). Overall, the model (Figure 2) suggests that teachers’ and healthcare workers’ community-focused and future-oriented attitudes complement parental engagement and optimism. Strengthening cross-sectoral collaboration among families, educators, and healthcare professionals may thus enhance patient satisfaction and support the psychosocial wellbeing of children with chronic illness.

## 4 Discussion

This study explored the attitudes of parents, teachers, and healthcare workers toward chronic illness in children receiving regular medical care. By examining these perspectives within a shared framework, the research highlighted both divergences and complementarities in the way these key reference persons approach chronic disease and its psychosocial implications. Importantly, attitudes toward chronic illness should be interpreted as contextual influences within the child’s psychosocial environment, rather than strictly causal determinants. Their role lies in shaping the relational and communicative climate in which parental satisfaction and child wellbeing emerge. Given the cross-sectional design of the study, the observed relationships should be interpreted as associative rather than causal. While the findings are consistent with theoretically expected directions, no conclusions regarding temporal or causal effects can be drawn.

Overall, the findings support the proposed integrated framework, suggesting that parental satisfaction and attitudes toward chronic illness operate as interconnected components within the child’s psychosocial environment. The findings revealed that parents demonstrate the strongest educational attitudes toward illness, which stem from their direct emotional and caregiving involvement with their child’s health. Their close personal connection often leads to greater concern, emotional strain, and heightened responsibility for disease management (33–37). However, parents’ perceptions of their child’s social inclusion and future prospects were often more negative than those of professionals, reflecting feelings of isolation and limited social support (38, 39).

Teachers exhibited the most positive peer relationship and community attitudes, emphasizing social integration, health education, and collective responsibility. Their everyday interaction with students and families positions them as mediators of information and support within the school environment (40). Teachers’ strong sense of community and optimism regarding the child’s future reflects their role in promoting resilience and wellbeing in educational contexts (41).

Healthcare workers also demonstrated positive attitudes, particularly in relation to vision and community, consistent with patient- and family-centered models of care. Their professional empathy, focus on prevention, and direct engagement in treatment foster constructive relationships that support children’s adaptation and quality of life (42, 43).

The proposed attitude model should be interpreted as an exploratory representation of observed associations rather than a confirmed system-level structure. Teachers’ peer and community attitudes were positively associated with their nurturing and visionary outlooks, while healthcare workers’ attitudes reinforced optimism and collaboration. Parental satisfaction was found to be associated with positive parenting attitudes and by supportive social and community contexts. Thus, shared commitment and consistent communication between parents, teachers, and healthcare professionals are essential to building a cohesive support network for children with chronic illnesses.

In addition, cluster analysis was used in an exploratory manner to identify potential patterns of parental satisfaction. While the cluster analysis provided a useful exploratory typology of parental satisfaction patterns, the small size of one subgroup (*N* = 5) suggests that these profiles should be interpreted with caution. The stability of cluster solutions may be sensitive to sample size and distribution, and the identified groups may not fully generalize to broader populations. Therefore, the clusters are best understood as indicative patterns rather than fixed or definitive categories. Alternative clustering approaches (e.g., hierarchical clustering or model-based methods) may yield different group structures, and future studies should examine the robustness of these patterns using complementary analytical techniques.

The results underline the need for stronger cooperation between the health and education sectors. Structured collaboration and communication among parents, teachers, and healthcare workers can reduce discrepancies in perception, strengthen mutual understanding, and enhance child-centered care. Interdisciplinary health promotion programs within schools can further improve attitudes toward chronic illness and promote long-term wellbeing.

## 5 Conclusion

This study provides an integrated perspective on the attitudes of parents, teachers, and healthcare professionals toward childhood chronic illness and provides preliminary insights into how these attitudes may be related to parental satisfaction and perceived child wellbeing. By examining these groups within a shared framework, the research highlights both role-specific differences and important points of convergence that shape the child’s psychosocial environment.

The findings indicate that parents exhibit the strongest educational engagement but also experience greater concern regarding social inclusion and future outcomes. In contrast, teachers and healthcare professionals demonstrate more positive community-focused and future-oriented attitudes, reflecting their roles in promoting inclusion, resilience, and long-term development. Importantly, positive attitudes—particularly those related to community support and future vision—are associated with higher parental satisfaction and more favorable psychosocial outcomes for children.

These results underscore the need for stronger, more structured cooperation between the health and education sectors. Consistent communication and shared responsibility among parents, teachers, and healthcare professionals can reduce discrepancies in perception, strengthen mutual understanding, and foster a cohesive support system for children with chronic illnesses. Interdisciplinary, school-based health promotion programs may further contribute to improving attitudes and enhancing long-term wellbeing.

Several limitations should be considered when interpreting the findings. The cross-sectional design precludes conclusions about causality or directionality, and it remains unclear whether attitudes influence parental satisfaction and child wellbeing, or whether these relationships operate bidirectionally. In addition, sociodemographic data were not equally detailed across all participant groups. Future research should involve larger and more diverse samples, incorporate longitudinal designs, and examine how sociodemographic factors and evolving interprofessional collaboration influence attitudes, satisfaction, and child outcomes over time. In addition, some subscales of the attitude measure showed only moderate reliability, which may have attenuated the strength of associations and limited the precision of the findings. In addition, the cluster analysis of parental satisfaction resulted in uneven group sizes, including one particularly small subgroup. This raises concerns regarding the stability and reproducibility of the cluster solution. Future studies with larger samples are needed to validate these profiles and to examine their robustness across different populations. In addition, no formal sensitivity analyses were conducted to test the stability of the clustering solution, which should be addressed in future research.

Despite these limitations, the present study contributes to a more nuanced understanding of how attitudinal dynamics across family, school, and healthcare contexts shape the experience of childhood chronic illness. Strengthening alignment among these key reference persons represents a promising pathway toward more effective, child-centered, and psychosocially responsive care.

## Data Availability

The raw data supporting the conclusions of this article will be made available by the authors, without undue reservation.
